# Evaluation of Thermo-Viscous Properties of Bitumen Concerning the Chemical Composition

**DOI:** 10.3390/ma16041379

**Published:** 2023-02-07

**Authors:** Eva Remisova, Dusan Briliak, Michal Holy

**Affiliations:** 1 Department of Highway and Environmental Engineering, Faculty of Civil Engineering, University of Zilina, Univerzitna 8215/1, 010 01 Zilina, Slovakia; 2 Zilina City Office, Namestie obeti komunizmu 1, 011 31 Zilina, Slovakia

**Keywords:** bitumen, dynamic viscosity, composition, elemental analysis, SARA

## Abstract

The quality of bitumen is standardized by conventional tests. With the development of new techniques, rotational and oscillatory measuring systems are applied to evaluate bitumen under defined geometric, temperature, frequency, stress, and strain conditions that correspond to loads during asphalt production and service. Several studies have focused on determining the effect of composition on bitumen properties at service temperatures. However, there is a lack of information related to the effect of composition on viscosity at higher temperatures, which influences production processes. The different types of bitumen, samples of 50/70, 35/50, 45/80-75, and 25/55-60 bitumen, had different viscosity values in intervals corresponding to a confidence level of 95%. The viscosity–temperature relationship in temperature range of 120 to 180 °C was observed in values of 3.87 and 3.70 for unmodified bitumen and 3.09 and 3.22 for modified bitumen. The effect of differences in SARA fractions content on the variation in viscosity using regression analysis showed the importance of asphaltenes (direct correlation) and aromates (negative correlation) contents for 50/70 bitumen with a coefficient of linear regression above 0.7. In comparison, the strong effect of saturates and asphaltenes (negative correlation) and resins was identified for 45/80-75 bitumen samples with correlation of 0.5 to 0.7.

## 1. Introduction

The viscoelastic character of bitumen predetermines its use in asphalt mixtures for the construction of road pavements. It is known that the properties of bitumen, specified by softening point and penetration, do not allow enough polymer or crumb-rubber modified bitumen to predict the service performance of the asphalt mixture [1,2]. Consistency at medium and elevated service temperatures and stability of consistency are evaluated without relation to bitumen composition and to stresses corresponding to the load in the real pavement [3]. The existing situation of asphalt pavements has resulted in the research for new methods and approaches to better describe the properties of bitumen binders and asphalts. One such approach is the so-called functional approach to assessing bitumen in terms of service performance. In the case of functional characteristics, the behavior is determined as a result of repeated stressing of the material under defined geometric, temperature and rate conditions using rotational and oscillatory measuring systems [4], or different forms and ranges of loading (temperature, frequency, stress) are chosen in which the bitumen binder is subjected to the effects under consideration. For the assessment of the properties of bitumen, modern methods are applied to better characterize the individual bitumen using rheological measurements (rotational, oscillatory, and creep tests) or deflection measurements.

At service temperatures (pavement serviceability of asphalt pavements), bitumen is highly viscous and its behavior is shown as a viscoelastic material. With their short time loading and low temperatures, binders behave elastic, with long time loading, viscoplastic strains occurring. In comparison, at higher temperatures (typically 60 °C above the softening point temperature) bitumen behavior corresponds to a flowing viscous liquid with a certain flow resistance (due to internal frictional forces between molecules) and is characterized by a temperature-dependent viscosity. For these non-Newtonian liquids, the viscosity is not a material constant (as a value of a ratio of shear stress and corresponding shear rate) but it is dependent on the degree and duration of the applied load.

The temperature dependence of bitumen viscosities is described by different mathematical models for different temperature regions. In the viscoelastic region, the Williams–Landers–Ferry (WLF) low equation is widely used to describe the temperature dependence of bitumen over a wide temperature range from bitumen glass-transition temperature Tg to Tg + 100 °C [5]. In work [6] the correlation was found between penetration and viscosity of paving grade bitumen based on rheometer measurements at the same temperature with validity over a range of temperatures, allowing the determination of penetration, less than the softening point. At high temperatures in the Newtonian region, the temperature dependence of low-viscosity liquids is described by the Arrhenius equation. The basic temperature-dependent viscosity equation has been modified many times to attempt to characterize non-Newtonian behavior. According to [7], the temperature dependence of viscosity at high temperature (typically superior to the softening point temperature) is generally described by the Walther equation prediction of the kinematic viscosity of crude oil products. The measurements of the viscosity of modified polymer bitumen viscosity by [8] showed the Saal equation viscosity–temperature relationship of fluids. Some research work later became the basis for future research with the aim of applying new methods and approaches for bitumen quality assessment.

Prediction of physical and rheological parameters and various testing methods need to be used to obtain relevant information. The performance of rotational tests is used to investigate more complex non-Newtonian flow behavior of liquids such as bitumen, which is important for the bitumen and asphalt processing (mixing, laydown, and compaction operations). The rotational viscometer can influence the shear rate and identify the behavior of Newtonian fluids (fluids that have viscosity independent of the shear rate) and non-Newtonian fluids (both pseudoplastic and dilatant behavior) [9]. Within the framework of various research activities [5,10,11] the thermoviscous properties of bitumen have been investigated in a rotational viscometer with the possibility of using the dynamic viscosity as a rheological characteristic expressing the structural–mechanical behavior of bitumen under the influence of strain forces as stated in [1].

The flow behavior of the material including bitumen is determined by its structure and chemical composition. Although according to the results of the SHRP program [5] it is very difficult to use chemical analysis to characterize the performance properties of asphalt due to the complexity and variation in the complex chemical structure, research has focused on investigating chemical parameters to describe the behavior of bitumen. Chemical composition of bitumen demonstrated by elemental analysis [12,13] depends primarily on the source of crude oil and processing technology. Petersen et al. [5] developed a linear combination to describe the bitumen viscosity on the basis of the content of the hetero atoms oxygen, sulfur, and nitrogen. With the development of new analytical techniques, the bitumen composition is expressed by group composition of chemically and structurally related compounds called SARA (Saturates, Aromatics, Resins, Asphaltenes) analyses [14,15].

Hofko et al. [16] emphasized the influence of SARA fractions and their interactions on the bitumen microstructure, and therefore on the viscoelastic behaviour. Study [17] of 70/100 paving grade bitumen microstructure showed an increase in asphaltene content that resulted in a decrease in creep compliance and therefore stiffer material behavior. Asphaltenes are the most studied bitumen fractions due to their considerable influence on the rheological properties and their viscosity-building role on [13]. The viscosity and stiffness modulus of bitumen increase with an increase in asphaltene content [18]. Concerning the temperature dependence of the viscosity, the asphaltenes have only a slight influence on it. As a consequence, bitumen with a low asphaltene content has a temperature-dependence close to that of their maltenes (which in turn will be close to that of lighter petroleum products), and high asphaltenes content possess higher activation energy for flow, meaning that they are less temperature susceptible [13]. For a growing content of saturates and aromatics, a reduction in stiffness as well as an increase in the viscous deformation behavior can be detected [19]. The growing contents of resins and asphaltenes cause an increase in stiffness, as well as an increase in the elastic deformation behavior of bitumen. Resin compounds also influence the temperature sensitivity of bitumen viscosity. Most of these studies [20,21] investigated the effect of composition on non-Newtonian behavior in the temperature range below the softening point temperature. Even with much research, the still unresolved areas provide scope for further research.

The research of bitumen thermoviscous behavior at the processing temperatures in relation to the composition of bitumen would help to explain the influence of bitumen composition on temperature sensitivity and processing temperatures in the production and laying of asphalt mixtures. Based on the above, this study investigates the possibility of the practical use of the dynamic viscosity measurements in the range of working temperatures for paving grade and modified bitumen to investigate thermoviscous properties as a qualitative parameter in relation to the composition of bitumen determined by means of elemental and compositional analysis.

## 2. Materials and Methods

### 2.1. Test Methods

#### 2.1.1. Conventional Testing of Bitumen

The physical properties of bitumen binders, which are in practice the most common indicators of their quality, were determined by conventional tests including the needle penetration at a temperature of 25 °C, and under specified conditions of load and loading duration according to EN 1426 [22] and a softening point test (the Ring and Ball Test method) according to EN 1427 [23] as the consistency at intermediate and elevated service temperatures.

#### 2.1.2. Viscosity Testing

The thermo-viscous behaviour of paving grade and modified bitumen samples was investigated using dynamic viscosity according to EN 13302 [24]. The basis of the dynamic viscosity measurement is to obtain a rate of the resistance of the sample against the applied stress, at a selected angular velocity. For the measurement of the bitumen viscosity, a rotational Brookfield viscometer model RVDV-II+Pro with controlled shear strain, thermoset system and spindle SC4-27 was used, based on the principle of rotation of the spindle, which is centrally immersed in the bitumen sample inside a second (static) cylinder (coaxial cylinder measuring system). The appropriate combination of spindle and speed produces satisfactory results in measurements from 10–100 on the instrument % torque scale. Functionality and reliability have been demonstrated by testing [25] with accuracy equivalent to that of glass capillary viscometers. The torque in the rotating cylinder (Searle principle method) is used to measure the relative resistance to the bitumen rotation at a certain temperature. The viscous properties of the bitumen were measured by the degree of spring reflection measured by a rotary transducer. The directly measured quantities in rotational viscometers are the angular velocity or the number of revolutions per time of steady motion of one of the cylinders, and data on the resistance of the fluid to shear stress due to the velocity gradient. The value of dynamic viscosity was determined using the rheological parameters of shear rate and shear stress. The measurements were performed at different temperatures (in the range of 120 to 190 °C), at which the consistency of the binder allows the measurement with respect to the range of the measuring instrument and spindle speeds (0.5 to 100 rpm). The results were recorded using Wingather V3.0 software. Previous research [26] has shown the shear rate and time at the selected temperature are important for viscosity measurement (period of 60 ± 5 s to stabilize the flow is not sufficient for low-viscosity materials or lower temperatures of 120 or 130 °C). Differences in the viscosity values were observed, caused by variations in the micellar structure of the bitumen owing to shearing in the measurement process. The time required to stabilize and obtain a constant value of dynamic viscosity, used in this experiment, was set to be 420 ± 5 s at a temperature of 120 to 130 °C, 300 ± 5 s at a temperature of 135 to 160 °C and 240 ± 5 s at a temperature of 135 to 190 °C.

#### 2.1.3. Elemental and Compositional Analysis

With the aim to analyze the impact of chemical composition on bitumen properties, the group and elemental composition were determined. Elementary Vario Cube with TCD detection equipment was used to determine CHNS content (in elemental analyze). The basic principle of quantitative determination of CHNS content is the combustion of a sample in the oxygen stream at high temperatures (up to 1200 °C). Gaseous combustion products (N_2_, CO_2_, H_2_O, and SO_2_) are purified, separated into individual components and analyzed on a TCD detector. The analyzed results include all combustible sulfur, organic and inorganic (S^2−^, SO_4_^2−^), as well as all combustible carbon, organically bound and inorganically bound (CO_3_^2−^). Gaseous combustion products are separated into individual components and analyzed using a thermal conductivity detector with an accuracy of <0.1%. This method is used to determine organic and inorganic sulfur and carbon, especially in petroleum products.

The determination of hydrocarbon-type chemical groups was performed by combining solvent extraction, and liquid adsorption liquid chromatography was used to characterize bitumen samples. The process started to separate the asphaltenes, 50 mL of n-heptane was added to 1,2 g of the bitumen sample with stirring. Sample dissolution was supported by ultrasound. Precipitated asphaltenes were filtered and extracted in a Soxhlet with hot heptane to remove oily substances, paraffins and ceresins precipitated together with asphaltenes. Soluble non-asphaltene fractions of bitumen (maltenes) were subjected to a further fractional procedure. Column chromatography was used for the separation of maltenes into saturates, aromates and resins. It involves passing maltenes in a weak solvent through a glass column of highly absorbent material. The sorbents were alumina 507C (Sigma Aldrich, St. Louis, MO, USA, activated for 8 h at 400 °C) and silica gel 60 (Fluka 0.063–0.2 mm, activated for 16 h at 160 °C) impregnated with silver nitrate. The resins were separated into alumina and toluene as the mobile phase. The aromatics were separated from the saturated hydrocarbons on silver nitrate-modified silica gel and n-heptane as the mobile phase.

### 2.2. Materials

The data presented in this paper were obtained from experimental measurements. Samples of paving-grade bitumen of 50/70 gradation (12 different samples) and 35/50 gradation (3 different samples) according to EN 12591, and polymer-modified bitumen 45/80-75 (5 different samples) and 25/55-60 (2 samples) in accordance with EN 14023 commonly used in practice for construction, repair and reconstruction of asphalt pavement layers in the Slovak Republic. The tested binders were obtained from different producers (MOL, Szazhalombatta, Hungary; OMV, Schwechat, Austria; Orlen and Lotos, Plock, Poland; Orlen, Litvinov and Total, Prague, Czech Republic).

## 3. Results and Discussion

### 3.1. Conventional Properties of Bitumen

**Figure 1:**
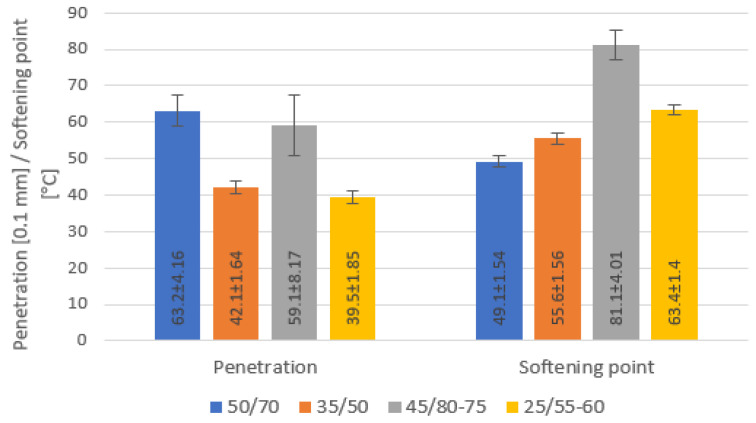
Penetration at 25 °C and softening point of tested bitumen samples.

The average values of the physical properties of all bitumen samples according to gradation are presented in Figure 1. Penetration of a bitumen sample, as consistency, was measured according to the European standard EN 1426 by determining the depth of penetration of a standard needle into the sample under known conditions of loading, time, and temperature. The softening point (the temperature at which the bitumen in the ring softens enough to allow a ball to fall at a distance of 25 mm) was determined according to EN 1427. Empirical properties showed that the values of the penetration and softening points values of all tested bitumen were in ranges specified in European standards and specified by the producers. The bitumen 50/70, 35/50, 45/80-75 and 25/55-60 were characterized by penetration values of 54.3 to 70.0 × 0.1 mm, 40.8 to 44.4 × 0.1 mm, 51.6 to 74.3 × 0.1 mm, and 37.6 to 41.3 × 0.1 mm, respectively, and by softening point values of 47.0 to 53.0 °C, 53.5 to 57.3 °C, 75.4 to 86.0 °C, and 62.0 to 64.8 °C and proved the expected difference in these values.

### 3.2. Elemental and Compositional Analysis

In relation to physical test methods to determine the basic characteristics and performance-related bitumen properties, the elemental and group compositions were determined. The aim was to identify differences in CHNS and SARA content of the bitumen samples that could explain the differences in the thermoviscous behavior of bitumen. Based on current knowledge and the results of a conventional tests, the difference in the composition among the bitumen was expected.

**Figure 2:**
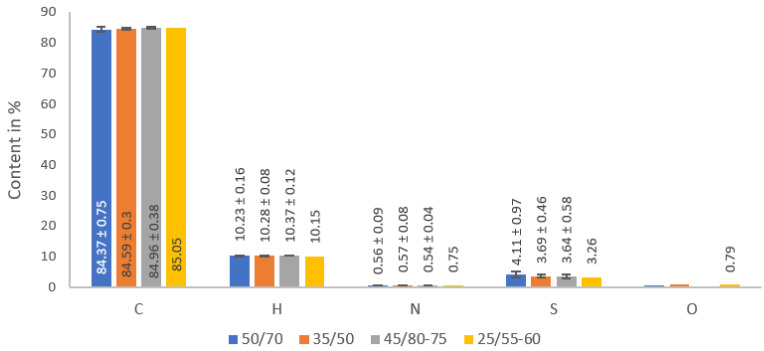
Elemental analysis (CHNS and O content) results of tested bitumen samples.

The average values and standard deviations of the CHNS and O content of the bitumen samples are presented in Figure 2. Regarding the elemental composition, no significant differences in the content of individual elements in the paving grade and modified bituminous binders were detected. This result is confirmed by the statistical analysis, for no one evaluated element the *p*-value was recorded less than the α (*p*-value 0.38 for carbon, 0.37 for hydrogen, 0.23 for nitrogen, and 0.63 for sulfur elements). The chemical composition of bitumen is extremely complex and varies by crude oil sources, production process and chemical modification similar to the findings in [4], and this affects the values determined for individual bitumen samples from different production batches.

**Figure 3:**
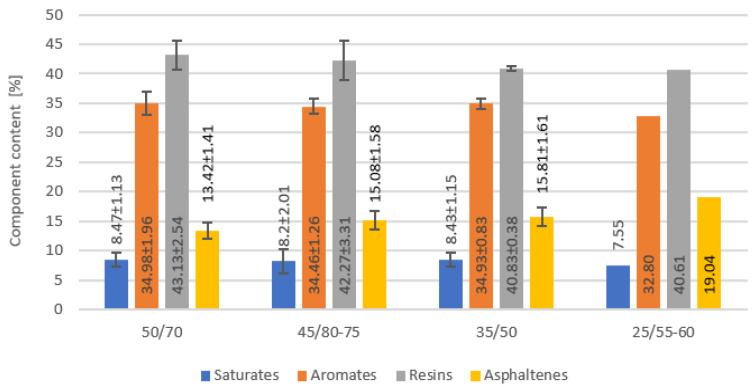
Fractional composition (SARA analysis) of tested bitumen samples.

Bitumen as a crude oil product consists of tens of thousands of different hydrocarbon molecules, and the properties of bitumen vary with their constitution. Hydrocarbon group analysis is commonly employed with the knowledge of the distribution of major structural groups of hydrocarbons according to the polarity. The SARA analysis showed some differences between bitumen (Figure 3), but they are not very significant. A smaller difference in fraction content was observed between particular samples of the same bitumen gradation type for 50/70 paving grade bitumen samples and modified bitumen samples 45/80-75, with the numbers of bitumen samples from more than one producer, different type of crude oil, production technology and production batches. Based on a statistical analysis of the impact of the fractional composition on binder differentiation in terms of different types of binder, the asphaltene content was shown to be relevant (*p*-value 0.006). The other three hydrocarbon groups showed statistical insignificance (*p*-value > 0.54). The highest content of asphaltenes as a key bitumen component that gives strength and stiffness to bitumen and is responsible for structure in bitumen was recorded for 25/55-60 bitumen, which corresponds to higher stiffness values (lower penetration, Figure 1). According to [27], saturates and aromatics act as a diluting medium and are responsible for bitumen viscosity and fluidity of the bitumen, which should appear in the viscosity values. The average values of saturates and aromatics are similar except for the 25/55-60 bitumen sample. 

**Figure 4:**
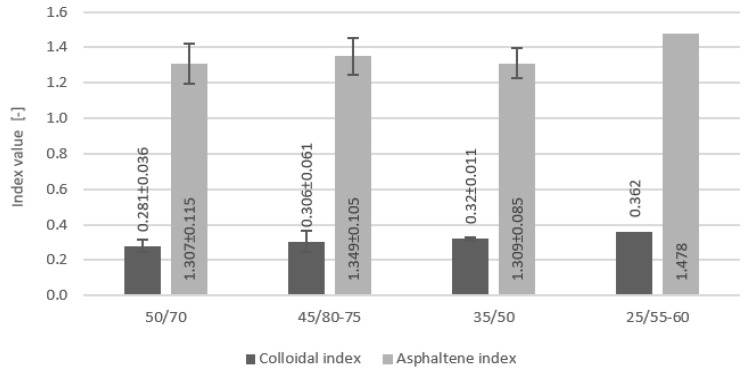
Composition indexes I_A_ and I_C_ on the base of SARA fractions of tested bitumen samples.

From the contents of the four SARA fractions, the single parameters, the Gaestel index and the asphaltene index [28], are calculated and used to describe specific bitumen characteristics. The colloidal instability index I_C_ is used to characterize the residual stability of bitumen, and the asphaltene index I_A_ expresses the change rate of asphaltene content. The calculated values of the indexes are shown in Figure 4. According to the colloidal instability requirements presented in [29,30], the higher the Gaestel index (from 0.5 to 2.7), the less stable the system. In addition, bitumen with Ic less than 0.22 becomes softer.

### 3.3. Thermo-Viscous Bitumen Properties

**Figure 5:**
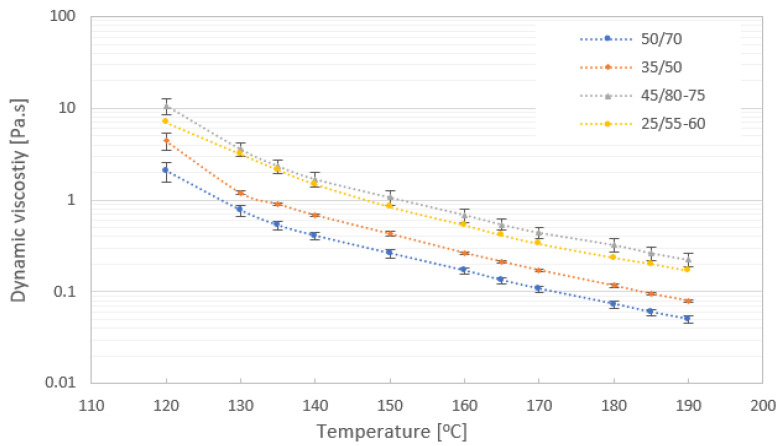
Dynamic viscosity results of tested bitumen samples 50/70 gradation, 35/50 gradation, modified bitumen samples 45/80-75 and 25/55-60.

The ability of bitumen to resist mechanical effects and its structural mechanical behavior under the influence of various performing forces depends on the viscosity of the material. Viscosity is the material property that relates the viscous stresses in a material to the rate of change in deformation (the strain rate). To compare viscosity data, the same test methodology was used: the same instrument, spindle, container, temperature, and test time for all test samples. The dynamic viscosity values of all tested bitumen samples at a temperature in the range of 120 to 190 °C are shown in Figure 5. With an increase in temperature, the bitumen as a thermoplastic material becomes softer until it achieves fluid consistency. The viscosity of all binders decreased, but it was slowly and tends to be less sensitive to changes in temperature when it was higher than 180 °C due to the forces between atoms and molecules in the material structure. The dynamic viscosity values in the temperature range of 120 to 190 °C showed that different types of bitumen have different viscosity values and different types of bitumen binders can be distinguished. The graphical presentation of viscosity shows higher viscosity values of modified bitumen (samples PMB 45/80-75 and PMB 25/55-60) compared to paving grade bitumen (samples 50/70 and 35/50) and differences in curve slopes expressing the rate of change in dynamic viscosity with temperature change.

**Figure 6:**
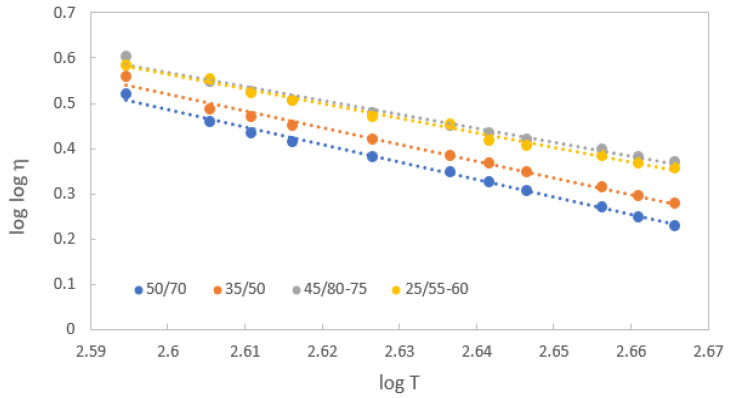
Viscosity–temperature relationship of tested bitumen samples.

The Saal equation [8] is the most commonly used model to describe the viscosity–temperature relationship for bitumen. This model was used to fit the dynamic viscosities of bitumen samples at different temperatures. The results of fitting are shown in Figure 6 and the corresponding parameters are shown in Table 1. According to [31], the slope of a linear model of a log–log viscosity and log–temperature graph defines the viscosity–temperature susceptibility VTS. The VTS values of tested 50/70 and 35/50 paving grade bitumen were determined in the range 3.71 to 4.11 and 3.64 to 3.73, respectively, and for modified bitumen samples 45/80-75 and 25/55-60 in the range 2.92 to 3.11 and 3.15 to 3.26, respectively. The slight slope of the modified bitumen expresses their less temperature susceptibility. This also shows that paving grade bitumen is more susceptible to viscosity changes with a temperature change compared to modified bitumen, and softer bitumen (with higher values of penetration and lower softening points) has lower viscosity values.

**Figure 7:**
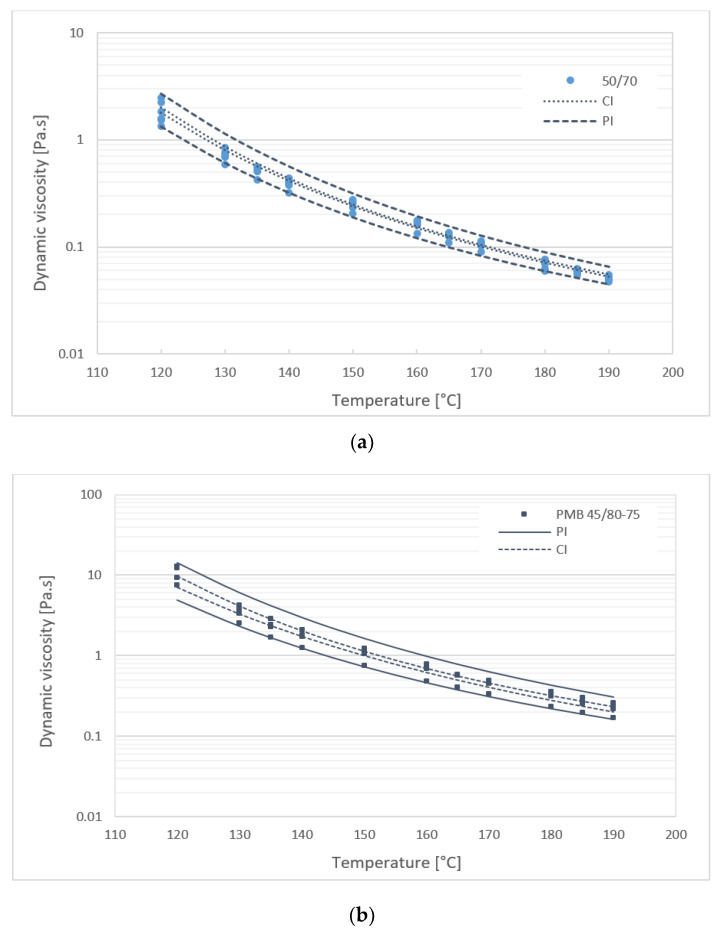
Dynamic viscosities of (**a**) 50/70 bitumen samples and (**b**) PMB 45/80-75 samples with confidence intervals.

Results of the measured dynamic viscosity in the temperature range of 120 to 190 °C have shown that different types of bitumen binders have different dynamic viscosity values. The dynamic viscosities of samples of the same bitumen type showed similarity between the values. This gives the possibility to define the viscosity limits at individual temperatures for various types of bitumen. The ranges of dynamic viscosity were determined using statistical prediction and confidence intervals that predict the value of a new observation based on the existing model, with a certain probability (1-α). The significance level α is set at 0.05 and corresponds to a 95% confidence level. Statistical intervals can be determined based on linear regression; therefore, the dependence log–log viscosity on log temperature was used to calculate. Figure 7 shows a 95% tolerance interval (confidence interval CI for average value and prediction interval PI for a single value) from the dynamic viscosity measurements of all samples of 50/70 paving grade bitumen and PMB 45/80-75 bitumen. Estimates represent intervals at which it is expected that the viscosity value at a certain temperature will be within this interval at 95% confidence.

**Figure 8:**
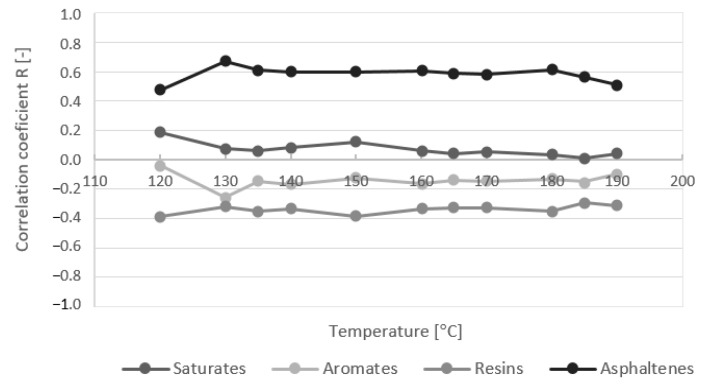
Example of correlation coefficient R of a linear model of dynamic viscosity and group composition at temperatures in the range 120 to 190 °C for group paving grade bitumen samples (50/70 and 35/50).

Statistical Anova analysis of the results of the dynamic viscosity in a temperature range of 120 to 190 °C showed that the temperature (expressed in steps of 10 °C and 5 °C, respectively) is a statistically significant element for viscosity values for paving grade bitumen binders 50/70 and the 35/50 gradation of bitumen and modified PMB 45/80-75 bitumen (result statistically significant *p* < 0.05). Similarly, Anova analysis (Table 2) confirmed the results shown in Figure 7 and Figure 8. For all temperatures evaluated in the range 120 to 190 °C, it was found that statistically significant differences were observed between the mean values of the dynamic viscosity of specific bitumen types (*p*-value < 0.001 between groups).

**Figure 9:**
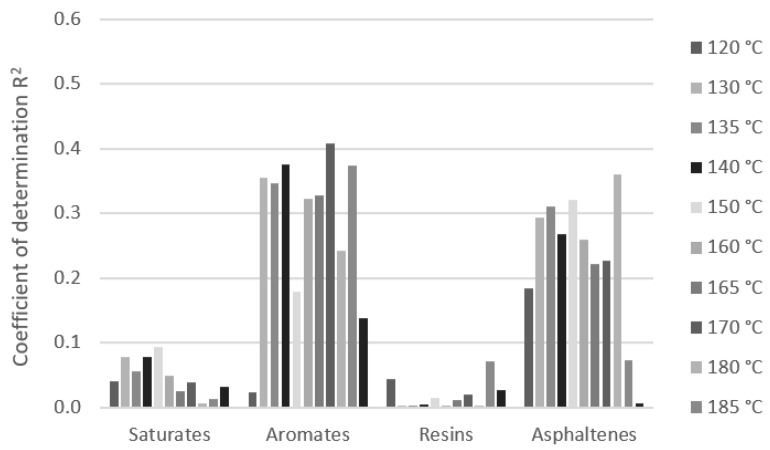
Coefficient of determination of linear model of dynamic viscosity and SARA composition at temperatures in the range 120 to 190 °C for group paving grade bitumen 50/70 samples.

**Figure 10:**
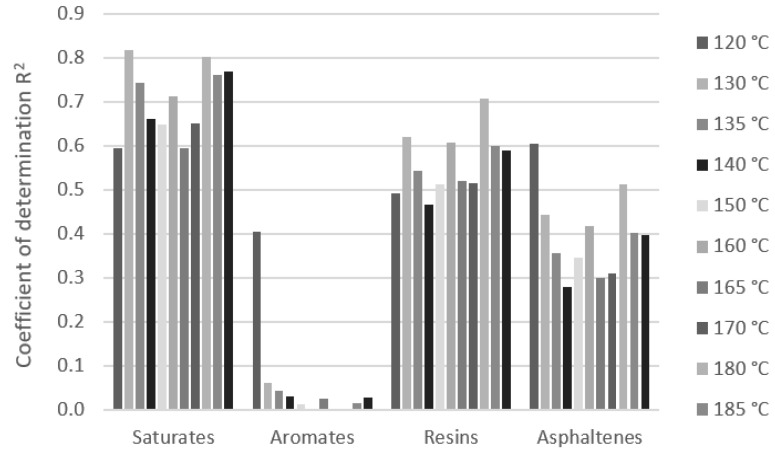
Coefficient of determination of linear model of dynamic viscosity and SARA composition at temperatures in the range 120 to 190 °C for group modified bitumen 45/80-75 samples.

**Figure 11:**
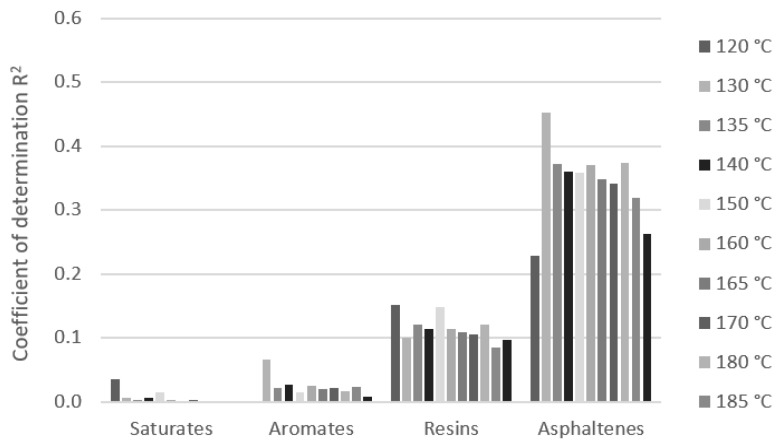
Coefficient of determination of linear model of dynamic viscosity and SARA composition at temperatures in the range 120 to 190 °C for group paving grade bitumen samples (50/70 and 35/50).

The chemical constitution has a significant effect on bitumen performance. The analysis of the effect of the SARA composition of the bitumen on its rheological properties expressed by dynamic viscosity was investigated. The dynamic viscosities of bitumen samples at particular test temperatures were used for regression analysis with the content of SARA fractions. The dependence was expressed by a simple linear regression model. The effect of the differences in the content of SARA fractions of bitumen on the variation in the values of the dynamic viscosity at all test temperatures was expressed by the coefficients of correlation and determination (Figure 8). The measurements of bitumen which had a sufficient number of samples were analyzed, and these were paving grade bitumen 50/70 samples Figure 9), modified bitumen 45/80-75 samples (Figure 10), and a group of paving grade bitumen 50/70 and 35/50, respectively (Figure 11). The R_2_ values varied in a certain range for analyzed bitumen samples. Moreover, the values for one fraction also varied in a small range of the test temperatures. 

Quantitative analysis of the composition of the saturated compounds, aromates, res-ins and asphaltenes fractions of samples 50/70 bitumen and bitumen viscosities based on Pearson’s correlation showed that as the asphaltenes content increases and decreasing the aromates content, an increase in viscosity was observed. The impact of the resin content was found to be small (negligible correlation ± 0.1) and the correlation coefficient of the saturates content was 0.1 to 0.3. Quantitative analysis of the fraction composition of paving grade bitumen samples (50/70 and 35/50 samples) showed that as the asphaltene content increases and the resins and aromates content decreases, an increase in viscosity was observed. The impact of the saturates content was found to be small (correlation 0.0 to 0.18). In the case of PMB 45/80-75 samples modified by polymer, the analysis showed that as the resins content increases and asphaltenes and saturates contents decrease, an in-crease in viscosity was observed. The composition of unmodified and modified bitumen with a similar gradation was shown to have a different impact on the viscosity values.

The significant effect of fraction content on dynamic viscosities is visible in the case of modified bitumen. The R_2_ values are higher than 0.7, which can be assessed as the strong effect of the saturates at several temperatures. The important effect could also be seen with the content of resins and asphaltenes (R_2_ values above 0.5). The R_2_ values for the aromates are relatively low and their effect on dynamic viscosity can be assessed as marginal. Regarding the content of the effect of the SARA fraction on dynamic viscosities for 50/70 paving grade bitumen samples, the content of aromates and asphaltenes could be important, although the R_2_ values do not exceed 0.4. No correlation was observed between the effect of resins and saturates content. When analyzing 50/70 paving grade bitumen samples together with 35/50, the effect of the asphaltenes content could be significant. The effect of the other three compounds (saturates, aromates, and resins) seems to be unimportant because of the low coefficient of determination. The R_2_ values are even much lower. These results are shown to be similar at all test temperatures in the range of 120 to 190 °C.

In this research, the dynamic viscosity at higher temperatures (which are relevant for the workability of bitumen during asphalt production and paving) was investigated and analyzed. These viscosities allow to set the appropriate temperature for mixing and compacting the asphalt mixture and thus characterize the workability of bitumen. In order to ensure good performance of the asphalt mixture, the temperature susceptibility of bitumen must be controlled [32].

## 4. Conclusions

The investigation was focused on the relationship between the composition (group) of bitumen identified SARA analysis and its thermoviscous properties determined in the rotational Brookfield viscometer. Fifteen samples of paving grade bitumen of two gradations and seven samples of polymer modified bitumen of two types were tested. The intention for the presented work was to investigate the influence of bitumen composition on its viscosity and the possibility of determining viscosity as an additional parameter for the assessment of binders. The following conclusions were summarized from the work:

- The elemental composition of tested bitumen showed no differences in the content of individual elements between paving grade bitumen 50/70 and bitumen 35/50 samples from different producers, or between paving grade and modified bitumen PMB 45/80-75; based on the SARA analysis the bitumen groups tested differ in asphaltene content from the average values of 13.4% for bitumen 50/70 to 19.0% for bitumen 25/55-60; although the composition results did not show significant differences between bitumen, the dynamic viscosity results in the temperature range of 120 to 190 °C demonstrated differences in values;
- The viscosity values of the bitumen samples of the same gradation at all evaluated temperatures in the range of 120 to 190 °C are similar and were in interval corresponding to a confidence level of 95%; the variations between the values are due to the different sources of crude oil and processing technology; different types of bitumen have different viscosity values (statistically significant differences between the mean values), the highest values were observed for PMB 45/80-75 and PMB 25/55-60, followed 35/50 bitumen and the lowest values of 50/70;
- The slope of the linear model expressing the viscosity–temperature relationship was observed as higher for paving grade bitumen 50/70 and bitumen 35/50, with a value of 3.87 and 3.70, respectively, compared to modified bitumen PMB 45/80-75 and PMB 25/55-60 with a value of 3.09 and 3.22; the SBS polymer has better compatibility with bitumen (aromates compound) and can influence viscosity;
- The analysis of the SARA fraction composition of the bitumen on viscosity values showed that the impact of asphaltenes and aromates content is important for paving grade bitumen 50/70 (coefficient of linear regression above 0.7), as the asphaltenes content increases and decreasing the aromates content, an increase in viscosity was observed; in comparison, the strong effect of saturates of modified bitumen 45/80-75 was identified (coefficient of 0.7); the important effect could also be seen with the content of resins and asphaltenes (coefficient above the 0.5).

The research presented does not cover the overall range of bitumen binders. Continued research and further dynamic viscosity measurements should lead to the setting up of databases and the objectification of limits. Then it will be possible to use the viscosity parameter to assess the quality of the bitumen.

## Figures and Tables

**Table 1:** Average values of fitted parameters for a viscosity–temperature model of 50/70 grade and 35/50 grade bitumen and modified bitumen 45/80-75 and 25/55-60.

Bitumen	Fitted Parameters		
	Slope	Offset	R^2^
50/70	3.873	10.556	0.996
35/50	3.697	10.085	0.991
45/80-75	3.093	8.611	0.988
25/55-60	3.219	8.931	0.992

**Table 2:** Analysis of variance for dynamic viscosity of the tested bitumen binders, α = 0.05, the effect of temperature.

Effect	50/70	35/50	PMB 45/80-75
	*p*-Value (d_f_ = 12)	*p*-Value (d_f_ = 3)	*p*-Value (d_f_ = 5)
Test temperature (in a range of 120 to 190 °C)	<0.001	<0.001	<0.001

## Data Availability

Data available on request.
